# Effects of Different Treatment Methods on the Clinical and Urodynamic State of Perimenopausal Women with Stress Urinary Incontinence

**Published:** 2018-08

**Authors:** Lifen LIU, Ying ZHANG, Jingya GONG, Xin CHEN, Hongmei WU, Weipei ZHU

**Affiliations:** Dept. of Gynaecology and Obstetrics, Second Affiliated Hospital, Soochow University, Suzhou 215004, PR China

**Keywords:** Perimenopause, Stress urinary incontinence, Urodynamics

## Abstract

**Background:**

This study was designed to investigate the clinical effects of different treatment methods on stress urinary incontinence (SUI) in perimenopausal women, and to evaluate urodynamic characteristics.

**Methods:**

Seventy-two menopausal female patients with stress urinary incontinence were included in the Second Affiliated Hospital of Soochow University from January 2016 to July 2017. The cases were divided into 3 groups of 24 each, depending on the treatment received. Group A patients received treatment with electrical stimulation combined with biofeedback; those in group B received conventional pelvic floor muscle exercise therapy; and those in group C did not have any treatment. Relevant clinical parameters of urination were determined including pelvic floor muscle strengths, urine dynamics indexes and ICS quality of life survey scores; results were averaged in each group for comparisons among the three groups before and after the 60-day study period.

**Results:**

After treatment for 60 days, both group A and B patients displayed a clear improvement in their urinary incontinence, pelvic floor muscle strength, leakage times, frequency of urination, urine dynamics index and ICS scores (*P*<0.05), with group A showing the most improvement. Women in group C showed no significant difference before and after the 60-day study period (*P*>0.05).

**Conclusion:**

Both the method of electrical stimulation combined with biofeedback, and conventional pelvic floor muscle exercises could help perimenopausal women with stress urinary incontinence; however, electrical stimulation combined with biological feedback seems to bring about better clinical effects.

## Introduction

Stress urinary incontinence (SUI) refers to the non-voluntary urine leakage from the urethra caused by sudden elevated abdominal pressure when coughing or sneezing. In China, the overall incidence of SUI in women is close to 20%. The disease occurs mostly in women aged 45–59 yr, in whom the incidence is more than 20% (1). The period of time before and after female menopause when the body is adjusting to the new state is known as the perimenopausal period. Perimenopausal women experience changes in their endocrine system and other relevant systems at varying degrees. SUI can seriously affect perimenopausal women’s physical and mental health and quality of life. The urinary kinetic parameters and pelvic floor muscle strength of patients with SUI show different degrees of abnormalities (2). Therefore, the treatment for SUI can be evaluated by measuring urodynamic parameters and pelvic floor muscle strength (3).

The current approach to clinical treatment of SUI is still surgery-based, but surgical treatment is traumatic, expensive and prone to postoperative complications. Therefore, there is a need for a safe and low-cost treatment approach. Biofeedback refers to a method in which recording of a specific physiological function of the patient by an instrument, is converted into acousto-optic information, so that the patient can learn to adjust the body according to the feedback signal. The degree of contraction in the pelvic floor muscles can be controlled by biofeedback (4, 5), which can be combined with electrical stimulation to restore pelvic floor function and prevent SUI. In addition, the pelvic floor muscle exercise method is also an effective way to control SUI (6).

This study aimed at comparing both these treatment methods, defining their effects on clinical and urodynamic values in order to help find an effective method for improving SUI in perimenopausal women.

## Materials and Methods

### General information

A total of 72 women with perimenopausal SUI were enrolled from the Second Affiliated Hospital of Soochow University from January 2016 to July 2017.

Inclusion criteria included female patients aged 40 to 60 yr, with intelligence in the normal range and diagnosed with perimenopausal SUI.

Patients presenting with neurogenic urinary incontinence, presenting other diseases, or having received treatment within the 7 days prior to their visit were excluded from the study.

In cases where a patient had to quit a group because of withdrawal from the trial or hospital transfer, a new patient was found in order to maintain the patient’s number in each group.

### Ethics approval

Subjects and family members signed informed consent forms to participate in the study. Researchers were responsible for protecting the safety of the subjects in the study, keeping the patient’s medical records confidential and protecting the privacy of subjects according to relevant principles of clinical guidelines.

Adherence to the double-blind clinical trial principles: researchers were divided into four groups: group 1 screened and distributed the subjects; group 2 enforced the treatment; group 3 observed and collected data; group 4 analyzed statistics and wrote the manuscript. The grouping of the subjects and the respective operations of each group were kept confidential.

### Grouping of subjects

The 72 patients enrolled were divided into three groups (A, B, and C), with of 24 cases in each. Patients in group A were treated with electrical stimulation and biofeedback. Group B patients were treated with conventional pelvic floor muscle training. And patients in group C did not follow any treatment. There were no significant differences in the average ages and disease courses for the three groups of SUI perimenopausal women (*P*> 0.05), so the data were all comparable (Table 1).

**Table 1:** General information of the subjects (n=24)

***Group***	***Age (yr)***	***Course (months)***
A	55.61 ± 6.07	24.45 ± 8.38
B	58.53 ± 8.15	27.02 ± 7.55
C	56.49 ± 6.88	27.54 ± 7.73
*F*	5.612	8.242
*P*	2.350	1.426

### Treatment methods

For the conventional pelvic floor muscle exercises the patients were instructed by professional supervisors to exercise their pelvic floor muscles for 30 minutes twice a week during 60 days. In the supine position, the patients were asked to perform a regular anal tightening movement and hold it for a few seconds, before completely relaxing the muscles and starting again, and repeating the procedure for 30 minutes.

The method of electric stimulation with biofeedback was performed using the PHENIX low frequency neuromuscular stimulation therapy instrument (U4 type, France). First, directed electrical stimulation (frequency: 50 Hz; pulse width: 250us; duration: 10 minutes) allows the patient to become aware of the muscles contracting; the next electrical stimulation (frequency: 8–32 Hz; pulse width: 320–740us; duration: 10 minutes) with biofeedback was used to train the type I muscle fibers. Second, the patients learned to differentiate abdominal from perineal contraction through electrical stimulation (frequency: 50 Hz; pulse width: 250us; duration: 10 minutes). Then electrical stimulation (frequency: 20 ~ 80Hz; pulse width: 20 ~ 320us; duration: 10 minutes) and biofeedback were used to train type II muscle fibers (frequency: 50Hz; pulse width: 250us; duration: 10 minutes).

The electrical stimulation and the A3 biofeedback scene-training module were given for 10 minutes. The above steps were repeated five times in a row. Patients received treatment twice per week during a period of 60 days. The patients were trained to have their pelvic floor muscles in a state of contraction when the abdominal pressure increased.

### Clinical Variables

The number of times urinary incontinence or urine leakage occurred, and the total number of urination episodes were counted and recorded by nurses, during the 60-day period.

The pelvic floor muscle strengths were measured using a low frequency neuromuscular therapy instrument (Guangzhou Cishan Company). The muscle potential probe was placed in the vagina of the patients and the other end was connected to DJZ-A and B low frequency neuromuscular therapy instrument to measure the changes in type I and II muscle fibers, which were then recorded. A classification was used to assess strength: the duration of the patient’s pelvic floor muscles contractions in seconds determined the classification of the muscle strength in grades (0, 1, 2, 3, 4, and 5 or more seconds of contraction determined grades 1, 2, 3, 4 and 5).

The urodynamic parameters before and after treatment were measured using a Life-Tech Janus-V urodynamic instrument (US Life-Tech) in accordance with international standardized operation procedures to determine the Valsalva leak point pressure (VLPP), the detrusor pressure at maximum urine flow (P_Qmax_), the maximum urethral closure pressure (P_MUC_), and bladder compliance (BC).

The Chinese version of the International Consultation on Incontinence Questionnaire-Urinary Incontinence Short Form (ICIQ-UI SF) was used before and after treatment, to obtain the age, gender, frequency of urinary incontinence, self-rated urine leak, the impact on the daily life of patients and the type of urinary incontinence. Items 3 to 5 were counted into the score to give a total score of 21 points. The lower the score, the better the urinary incontinence situation will be.

### Statistical methods

The data were all analyzed by SPSS20.0 statistical analysis software (IBM, USA). Metrological data were showed as “mean ± standard deviation” (*x̄* ± S). Data before and after treatment were generally analyzed using the paired t test. Comparisons between groups were conducted using variance analysis. Quantitative data were shown as percentages. Chi-square test was used for their analysis. P<0.05 for a given difference was considered statistically significant.

## Results

### Number of urinary incontinence episodes before and after 60-day study period for the subjects in the 3 groups

There were no significant differences in urinary incontinence among the three groups before treatment. After the 60-day study period, the incontinences of subjects A and B were improved significantly, and differed from those before treatment (*P*<0.01). There were no significant differences for group C individuals before and after the study period (11.71 ± 3.50 vs. 11.67 ± 3.19) (Table 2).

**Table 2:** Number of urinary incontinence episodes before and after the 60-day study period for patients in the three groups (n=24, *x̄* ± S)

***Group***	***Before***	***After***	***t***	***P***
A	11.61 ± 3.25	0.44 ± 0.25	12.359	0.002
B	11.72 ± 3.04	7.57 ± 2.84	9.623	0.015
C	11.67 ± 3.19	11.71 ± 3.50	2.314	0.412

### Pelvic floor muscle strength changes in individuals in the 3 groups after the 60-day study period

There were no significant differences in pelvic floor muscle strength among the individuals in the 3 groups before treatment. After 60 days of treatment, the pelvic floor muscle strengths of groups A and B increased to some extent, (P<0.02). Furthermore, the pelvic floor muscle strengths of group A individuals improved greater (patients in grades I to II initially were found in grades III–V after treatment).

The posterior pelvic floor strengths of individuals in group C showed no significant changes compared to the former assessments (Table 3).

**Table 3:** Pelvic floor muscle strength changes of the patients in the three groups before and after 60-day study period (n=24)

***Group***	***Grade I (n)***		***Grade II (n)***		***Grade III (n)***		***Grade IV (n)***		***Grade V (n)***	
	***Before***	***After***	***Before***	***After***	***Before***	***After***	***Before***	***After***	***Before***	***After***
A	16	0*	8	0*	0	4*	0	6*	0	14*
B	15	1*	9	7*	0	4*	0	6*	0	6*
C	15	16^#^	9	8^#^	0	0^#^	0	0^#^	0	0^#^
X^2^	1.957	9.318	2.101	8.692	1.000	8.957	1.000	8.240	1.000	9.254
*P*	0.323	0.000	0.245	0.000	1.000	0.010	1.000	0.005	1.000	0.001

*P* <0.05 (before treatment vs. after treatment) //

# *P*> 0.05 (before versus after 60-day period)

### Comparison of the number of urine leaks in individuals of the 3 groups before and after the 60-day study period

There were no significant differences in the number of urine leaks before and after the study period among the 3 groups. The number of urine leakage episodes in group A individuals was significantly improved after 60 days, and that number was significantly lower than that before treatment (2.33 ± 1.33 vs. 12.25 ± 2.75, *P*<0.05). The urine leaks in group B were also improved, although the degree of improvement was less than that in group A (6.67 ± 2.33 vs. 11.75 ± 3.30, *P*<0.01). The frequency of leakage in group C was slightly higher than that before treatment (11.75 ± 2.50 vs. 11.50 ± 3.75), but there was no statistical significance (Table 4).

**Table 4:** Comparison of the number of urine leaks of the patients in the three groups before and after 60-day study period (n=24)

***Group***	***Before***	***After***	***t***	***P***
A	12.25 ± 2.75	2.33 ± 1.33	7.853	0.004
B	11.75 ± 3.30	6.67 ± 2.33	6.665	0.027
C	11.50 ± 3.75	11.75 ± 2.50	2.010	0.378
*F*	2.117	29.365	-	-
*P*	0.315	0.001	-	-

### Comparison of the number of urination episodes of the individuals in the 3 groups before and after the study period

There were no significant differences in the number of urination episodes at the beginning of the study period in the 3 groups. The number of urination episodes in group A and B was significantly reduced after treatment (*P*<0.001). No significant differences were found in group C before and after treatment (33.02 ± 5.64 vs 33.06 ± 5.76, (Table 5).

**Table 5:** Comparison of the number of urination episodes of the patients in the three groups before and after 60-day study period (n=24)

***Group***	***Before***	***After***	***t***	***P***
A	33.07 ± 5.34	13.15 ± 2.66	7.312	0.003
B	33.85 ± 5.01	17.35 ± 3.28	9.257	0.001
C	33.02 ± 5.64	33.06 ± 5.76	1.961	0.425
*F*	1.964	22.510	-	-
*P*	0.385	0.012	-	-

### Comparison of changes in urodynamic indicators of the three groups before and after study period

At the beginning of the study, the measurement of urodynamic indicators showed no significant differences among the 3 groups regarding VLPP, P_Qmax_, P_MUC_ and BC. After 60 days of treatment, the VLPP, P_Qmax_, P_MUC_ and BC of groups A and B were significantly higher than those before treatment (*P*<0.01). Group C showed a slight decrease in the above indicators, but the difference was not significant (Table 6).

**Table 6:** Comparison of urodynamic indicators of the patients in the three groups, before and after 60-day study period

*Group Time*	*VLPP(cmH_2_O)*		*P_Qmax_ (cmH_2_O)*		*P_MUC_ (cmH_2_O)*		*BC (Ml/cmH_2_O)*	
	*Before*	*After*	*Before*	*After*	*Before*	*After*	*Before*	*After*
A	80.19 ± 6.21	110.34 ± 12.52*	40.04 ± 2.34	51.14 ± 4.26*	71.45 ± 5.41	91.22 ± 6.57*	40.00 ± 4.60	50.83 ± 6.22*
B	80.05 ± 6.25	96.21 ± 9.36*	40.23 ± 2.15	45.52 ± 3.81*	71.43 ± 5.71	83.58 ± 6.16*	40.19 ± 4.51	45.50 ± 5.24*
C	80.77 ± 5.81	78.06 ± 6.99^#^	40.37 ± 2.69	39.17 ± 3.36^#^	70.99 ± 6.81	70.56 ± 5.92^#^	40.33 ± 4.73	38.75 ± 5.11^#^
*F*	2.217	19.852	1.996	15.257	2.328	18.287	1.455	14.573
*P*	0.406	0.002	0.335	0.005	0.295	0.002	0.354	0.007

*P* <0.05 (before treatment vs. after treatment)

# *P*> 0.05 (before treatment vs. after 60-day study period)

### Comparison of changes in ICS scores of the individuals in the 3 groups before and the 60-day study period

There were no significant differences among the three groups in ICS scores before treatment. After 60 days of treatment, the ICS scores of groups A and B were significantly higher than those before treatment (*P*<0.01). Group C showed slightly worse scores compared to the beginning of the study, but the difference was not significant (Table 7).

**Table 7:** Comparison of ICS scores of patients in the three groups, before and after 60-day study period (n=24)

*Group Time*	*Score: 0–1 (%)*		*2 (%)*		*3–4 (%)*	
	*Before*	*After*	*Before*	*After*	*Before*	*After*
A	0.0.00	20.83.33*	9.37.5	4.16.67*	15.62.50	0.0.00*
B	0.0.00	8.33.33*	9.37.50	7.29.17*	15.62.50	9.37.50*
C	0.0.00	0.0.00^#^	10.41.67	9.37.50^#^	14.58.33	15.62.50^#^
X^2^	1.000	8.58	2.320	6.667	3.002	10.653
*P*	1.000	0.000	0.216	0.035	0.197	0.000

* *P* <0.05 (before treatment vs. after treatment)

# *P*> 0.05 (before treatment vs. after 60-day study period)

## Discussion

SUI refers to the involuntary leakage of urine due to increased abdominal pressure when patients laugh, sneeze or cough (7). 45–59 is the age where the incidence of SUI peaks in women (8). SUI patients often present abnormalities in urodynamic indicators and pelvic floor muscle strength (9). Therefore, changes in urodynamic indicators and pelvic floor muscle strength in SUI patients can be used to evaluate and determine the clinical effects of treatment (10, 11). The most common approach to non-surgical SUI treatment is pelvic floor muscle training. Although this method alleviates the symptoms of the disease, its effects are not completely satisfactory. Electrical stimulation combined with biofeedback is a relatively new treatment method, which evaluates the patient’s muscle function and guides the following recovery training through biofeedback (12, 13). Although there have been studies on treating patients with abnormal pelvic floor muscle strength using biofeedback, the results have not been consistent (14–16).

This study showed that, after 60 days of treatment, the urinary incontinence, pelvic floor muscle strength, number of leakage episodes, frequency of urination, urine dynamics index and ICS scores for groups A and B had significantly improved. Group A, which used the treatment of electrical stimulation combined with biofeedback, showed the most improvement. Group C, which received no treatment, showed no significant differences before and after the study period. This highly suggests that electrical stimulation with biofeedback and conventional pelvic floor muscle training alone are clinically effective to different degrees, with the former treatment giving better results than the latter one. One explanation is that the conventional pelvic floor muscle training is not as precise causing patients to increase the function of many different muscles, therefore inadvertently increasing the maximum urethral closure pressure (P_MUC_).

Electrical stimulation combined with biofeedback may be more effective in improving the pelvic function and urodynamic indicators of patients (10, 17). Electrical stimulation combined with biofeedback can transmit currents of different pulse width and intensity to stimulate the pelvic floor muscle, which increases the elasticity and strength of the muscle, therefore controlling the urine leaks to some extent (18). In addition, electrical stimulation combined with biofeedback inhibits detrusor muscle excitability and detrusor contraction by stimulating the fibers of the pudendal nerve. When the electrical stimulation impulse moves up to reach the thoracolumbar region, α-adrenergic receptors can promote contraction of the urethral proximity and the bladder neck, thereby enhancing the function of urethral closure. Also, excitement of the β-adrenergic receptors can enhance the bladder neck closure by relaxing the bladder bottom (19–21).

In our experience, the ICS scores of groups A and group B increased significantly after 60 days of treatment. The scores of subjects in group A showed a more obvious increase. Group C showed slightly worse scores but the difference was not statistically significant compared to the values at the beginning of the study period. Our results, suggest that two treatments improved not only the clinical parameters of the SUI in perimenopausal women, but also the quality of life of those affected.

## Conclusion

Both the method combining electrical stimulation and biofeedback and the conventional pelvic floor muscle training are helpful for the improvement of SUI in perimenopausal women; however the effects of the electrical stimulation combined with biofeedback seem to be superior and warrant further studies in order to make a general recommendation.

## Ethical considerations

Ethical issues (Including plagiarism, informed consent, misconduct, data fabrication and/or falsification, double publication and/or submission, redundancy, etc.) have been completely observed by the authors.
